# The Codelivery of siRNA and QDs by pH-Responsive Micelle for Hepatoma Cancer Cells

**DOI:** 10.3389/fphar.2019.01194

**Published:** 2019-10-10

**Authors:** Zhonglin Cao, Huiyu Xiao, Li Li, Maixian Liu, Guimiao Lin, Peng Zhai, Ken-Tye Yong, Xiaomei Wang, Gaixia Xu

**Affiliations:** ^1^Key Laboratory of Optoelectronics Devices and Systems of Ministry of Education and Guangdong Province, College of Optoelectronic Engineering, Shenzhen University, Shenzhen, China; ^2^Guangdong Key Laboratory for Biomedical Measurements and Ultrasound Imaging, School of Biomedical Engineering, Health Science Center, Shenzhen University, Shenzhen, China; ^3^Department of Physiology, School of Basic Medical Sciences, Shenzhen University Health Sciences Center, Shenzhen, China; ^4^School of Electrical and Electronic Engineering, Nanyang Technological University, Singapore, Singapore

**Keywords:** siRNA, QDs, micelle, gene carrier, hepatoma cancer cell

## Abstract

Recently, RNA interfering (RNAi) has become a promising approach for cancer therapy. However, the application of RNAi for clinics is still hindered due to the lack of safe and efficient carriers. In this study, a pH-responsive micelle based on polycaprolactone-block–poly 2-(dimethylamino)ethyl methacrylate (PCL-PDEM) cationic copolymer was developed to carry short interfering RNA (siRNA) for silencing interleukin 8 (IL-8) gene in hepatoma cancer cells. The transfection efficiency of the PCL-PDEM-siRNA/quantum dots (QDs) nanoplex has reached about 70%, and the expression level of IL-8 decreased about 63%. Furthermore, the codelivery of QDs and siRNA has been realized, which is beneficial to visualize the process of siRNA delivery. No considerable cytotoxicity from the nanoparticles has been observed, indicating that our responsive cationic micelle is potential in clinical trial for hepatoma cancer therapy.

## Introduction

As one of the most deadly and the second most common cancers throughout the world, hepatoma cancer is estimated to claim 750,000 lives every year worldwide, 51% of which occur in China (Wang et al., 2014). Although the diagnostics and therapy of hepatoma cancer have been developed, many challenges remain to be tackled collaboratively.

RNA interference (RNAi) is a promising technique with great opportunity to cure cancer due to its revolutionary therapeutic strategy and high specificity (Mansoori et al., 2014). The invention of RNAi has brought promises for gene therapy of cancer by using small interfering RNA (siRNA), which selectively silences specific targets (Seyhan, 2011). In the past few years, RNAi-based cancer gene therapy has been extensively explored (Lin et al., 2013; Maduri, 2015; Gan et al., 2016; Lin et al., 2017). However, the safe and efficient delivery carrier are critical barriers for successful RNAi gene delivery and therapy. The naked siRNA is subjected to degradation by RNase. What’s more, free siRNA is unable to penetrate the cell membrane due to its negatively charged nature (Whitehead et al., 2009). To address these issues, the researchers developed a great deal of carriers to delivery siRNA into cytoplasm (Lin et al., 2013; Shi et al., 2014; Dim et al., 2015; Li et al., 2015; Fitzgerald et al., 2016; Warashina et al., 2016). The viral vectors possessed high transfection efficiency, as well as low production, high cost, and potential biosafety issues (Marshall, 1999). Recently, nonviral carriers, especially cationic polymers, have drawn attentions due to many advantages comparing with viral vectors, including improved safety, low immune responses, and stimuli-responsive functionality (Whitehead et al., 2009; Zhu et al., 2010).

Besides RNase-induced degradation, lysosomal degradation is another issue hampering the effective delivery of siRNA into cytosol. It was known that endocytosis is followed by the fusion of siRNA-complexed nanocarriers with the early, mildly acidic endosomes, which transform into late endosomes. Lysosomes are the final organelles involved in the endosomal degradation pathway, in which nucleic acids and proteins are hydrolyzed. (Savic et al., 2003; Amjad et al., 2017) This implied that nanocarriers should be designed with the ability of endosomal escape to release siRNA before subcellular degradation. It has been found that the pH value is decreasing from endosome to lysosome after endocytosis. Typically, the pH of lysosomes is about 4 to 5, and that of late endosomes is near 5 (Schmid et al., 1988). Therefore, the pH decrease in endosomal pathway should be considered in materials design for siRNA delivery. The acidity of the tumor extracellular fluid was used as a trigger to generate positive charges (Xu et al., 2007) or expose the targeting groups (Lee et al., 2005) on the nanoparticle surface to promote endocytosis for fast cellular uptake. The pH decrease of endosomes could induce nanoparticles to regenerate positive charges and disrupt the endosomal membrane for nanoparticle escape (Xu et al., 2007). In addition, polymers with amino groups could disrupt acidifying endosomes via the “proton sponge” effect (Hu et al., 2007) and release siRNA into cytoplasm.

The growth of tumor was sustained by oxygen and nutrition supplied from angiogenetic blood vessels (Aalinkeel et al., 2004). Angiogenesis was a feature of aggressive tumors, which was regulated by stimulators and inhibitors of the proliferation, migration, and invasion of endothelial cells (Liotta et al., 1991). Various growth factors could induce angiogenesis, such as interleukin 8 (IL-8), vascular endothelial growth factor, transforming growth factor β, and tumor necrosis factor α (Folkman and Klagsbrun, 1987). IL-8 was secreted by various stromal cells (e.g., endothelial cells and fibroblasts) and cancer cells (e.g., hepatoma, melanoma, prostate cancer, and endometrial cancer). IL-8 also modulated the survival and proliferation of tumor cells of hepatoma, prostate, mammary, and gastric cancers (Chen et al., 2012). In addition, increasing of IL-8 levels was believed to promote the metastasis of cancer cells as well (De Larco et al., 2003). It was reported that the IL-8 levels in serum of patients contracting hepatoma cancer upregulated the level of IL-8 (Welling et al., 2012). Therefore, the instrumental role of IL-8 in tumor aggression implied that blocking its expression could be an efficient mean of treatment.

The fluorescent quantum dots (QDs) have unique optical features such as high resistance to photobleaching, size-tunable fluorescent peaks, and a broad excitation profile with narrow emission spectra (Xu et al., 2016). The exceptional optical properties of QDs make them useful for real-time monitoring of the siRNA delivery process* in situ*. The cationic micelles are particularly appealing because they have the potential to simultaneously deliver siRNA and hydrophobic QDs.

**Scheme 1:**
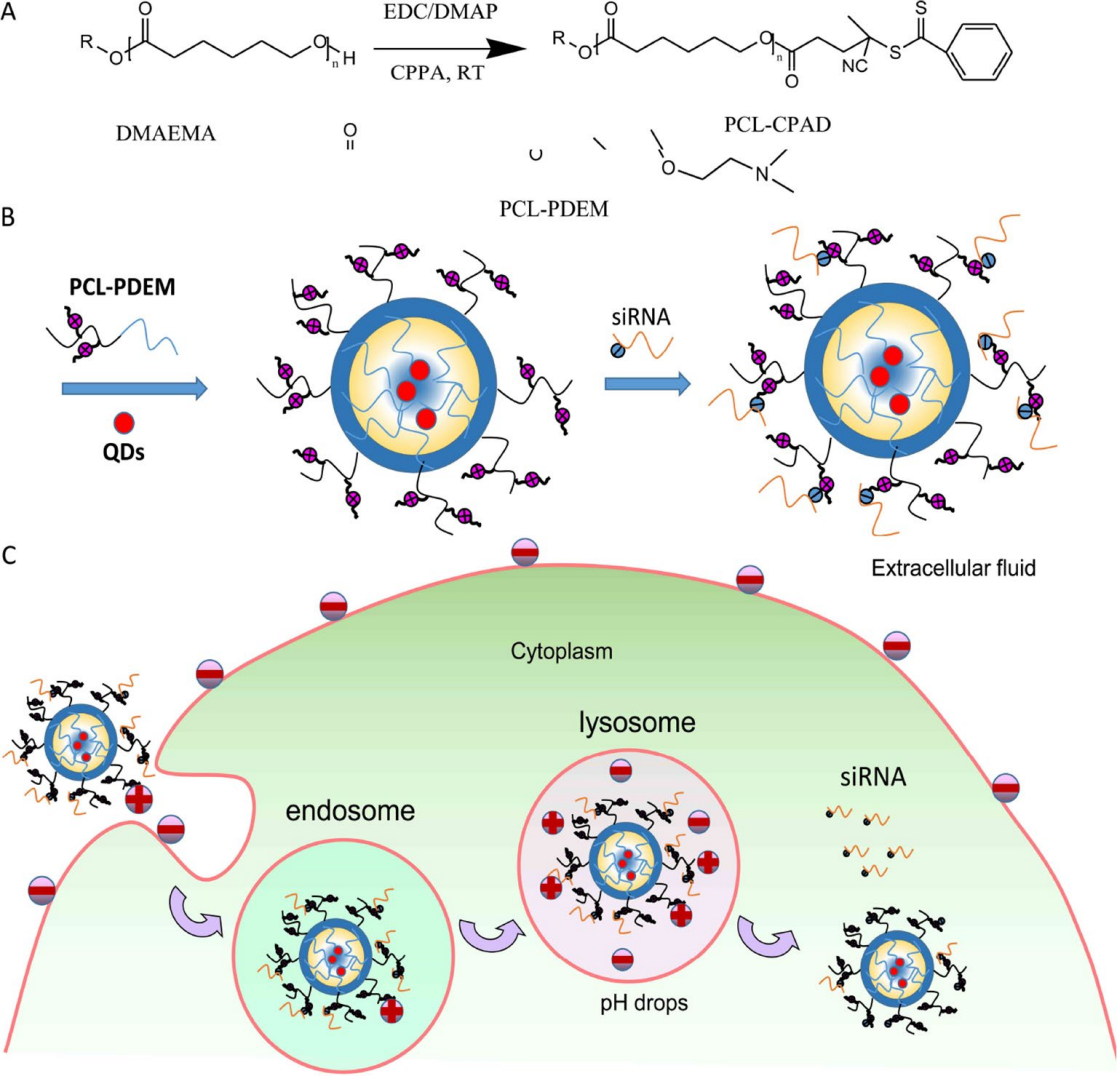
**(A)** Synthesis of PCL-PDEM copolymers. **(B)** The scheme of the PCL-PDEM-siRNA/QDs nanoplexes. **(C)** The scheme of delivery nanoplexes into cancer cells.

In this study, the copolymer constructed with pH-responsive poly 2-(dimethylamino)ethyl methacrylate (PDEM) and biodegradable polycaprolactone (PCL) was used as nanocarriers to effectively delivery siRNA into SK-Hep1 cells for gene silencing of IL-8 (as shown in **Scheme 1A**). The delivery efficacy of nanocarriers was comparable to that of the commercially available Lipofectamine, and the expression of IL-8 is successfully suppressed. The cytotoxicity study further revealed that the nanocarriers were noncytotoxic. QDs were encapsulated in nanocarriers, which codelivered the QDs and siRNA. This work may help to establish pH-responsive micelle for siRNA delivery and imaging, which would be a useful nanoplatform for extensive future studies in liver cancer therapy.

## Materials and Methods

### Materials

1-(3-Dimethylaminopropyl)-3-ethylcarbodiimide hydrochloride (EDC), 4-dimethylaminopyridine (DMAP), 4-cyanopentanoic acid dithiobenzoate (CPAD), azodiisobutyronitrile (AIBN), and PCL-OH (*M*_n_ = 5000, polydispersity [PDI] = 1.1) were purchased from Sigma and used as received. 2-(Dimethylamino) ethyl methacrylate (DEM, Sigma) was purified by passing through a basic alumina column before use. The tetrahydrofuran (THF) and dichloromethane were dried by refluxing over sodium wire and CaH_2_, respectively, and distilled prior to use. CdSSe/ZnS QDs (5 mg ml^−1^ in toluene solution) were purchased from Najingtech Company.

Dulbecco modified eagle medium (DMEM), fetal bovine serum (FBS), trypsin, penicillin, and streptomycin were purchased from Gibco. IL-8 enzyme-linked immunosorbent assay (ELISA) kit and MTS were purchased from Invitrogen. IL-8 siRNA^FAM^ (sense: 5′-FAM- GGAUUUUCCUAGAUAUUGCdTdT-3′; antisense: 5′- GCAAUAUCUAGGAAAAUCCdTdT-3′) was purchased from Genepharma Company, China.

### Synthesis and Characterization of Stimuli-Responsive Polymer

The stimuli-responsive copolymers PCL-PDEM were synthesized by reversible addition fragmentation chain transfer (RAFT) polymerization (**Scheme 1B**). First, the chain transfer agent was grafted onto the end of PCL with an *M*_n_ = 6,318 Da and PDI = 1.16. EDC (0.72 g, 3.6 mmol) and DMAP (0.14 g, 1.2 mmol) were dissolved in dichloromethane (10 ml) and added into a 100-ml round-bottomed flask equipped with a magnetic stirring bar. CPAD (0.27 g, 0.99 mmol) was dissolved in dichloromethane (5 ml) and added to the above mixture in portions. The solution was stirred moderately for 15 min, after which PCL-OH (2.25, 0.45 mmol), dissolved in dichloromethane (10 ml), was added dropwise. After stirring at room temperature for 3 days, the reaction mixture was precipitated into excess cold diethyl ether three times. The pink-colored PCL-CPAD was obtained and dried in vacuum for 24 h. ^1^H NMR (400 MHz, CDCl_3_, δ): 7.9 to 7.4 (Ar*H* in CPAD end group), 4.1 (-C*H*_2_COO-), 3.6 (-C*H*_2_OCO-), 1.9 to 2.3 (-C*H*_2_-), and 2.6 (-C*H*_3_ in CPAD end group). A typical copolymerization was performed as follows. A 20-ml glass ampule was charged with AIBN (3.2 mg, 0.02 mmol), PCL-CPAD (0.58 g, 0.088 mmol), DEM (0.70 g, 4.4 mmol), THF (10 ml), and a magnetic stirring bar. The ampule was flame sealed under vacuum after three freeze-thaw cycles and placed in a thermo-stated oil bath at 70°C for 24 h. After the polymerization, the ampule was immersed in liquid nitrogen to quench the polymerization, and the polymer (PCL-PDEM) was isolated by precipitation in diethyl ether. The precipitation was repeated three times, and the product was dried in vacuum at room temperature overnight (yield: 99%). ^1^H NMR (400 MHz, CDCl_3_, δ): 4.1 (-C*H*_2_COO-), 3.6(-C*H*_2_OCO-), 1.8 (-N*H*_2_), 2.5 (-NC*H*_3_), 1.0 (-C*H*_3_), and 1.25 (-C*H*_2_). The molecular weight of polymer was analyzed to be 17,195 Da and the PDI to be 1.66 via gel permeation chromatography (GPC) (Alliance e2695; Waters, Singapore). The chemical structure of the PCL-PDEM is shown in **Scheme 1**.

### Preparation and Characterization of Nanoplex

Five milligrams PCL-PDEM and 0.5 mg QDs were dissolved in 0.5 ml THF. The solution was added dropwise into 5 ml 0.01 M phosphate-buffered saline (PBS) buffer solution (pH 7.4). After stirring at room temperature for 24 h, the micelle solution (PCL-PDEM/QDs) was obtained. The hydrodynamic sizes and the zeta potentials of micelles were determined at 25°C by Zetasizer Nano ZS (Malvern Instruments). The fluorescence spectra of CdSSe/ZnS QDs were determined by a spectrophotometer (F-4600; Hitachi, Japan). The morphology images of the particles were obtained with a transmission electron microscope (TEM) (Tecnai G2 F20 S-TWIN; FEI, USA) operating at an accelerating voltage of 200 kV at room temperature. The resulting complexes were electrophoresed on a 1.2% agarose gel at 100 V for 10 to 15 min using GelRed as nucleic acid dyes. Subsequently, the gel was observed and imaged under a UV transilluminator (Bio-Rad).

### The SiRNA Release From Nanoplex

The siRNA release studies were carried out in PBS (pH 7.4, 10 mM) and 37°C using a dialysis tube (MWCO 100000) in the presence of poly(aspartic acid) as model polyanion. The PCL-PDEM/siRNA nanoplexes with P/N = 0.1 (siRNA concentration was 4.4 µM, and micelle concentration was 0.55 mg/ml) were prepared. To acquire sink conditions, the siRNA release studies were performed with 0.8 ml of nanoplexes solution dialysis against 5.0 ml of the same media. According to the molar ratio of aspartic residue to siRNA phosphate of 10, 0.2 ml poly(aspartic acid) solution (0.80 mg/ml) was added. At desired time intervals, 10 µl of release media was taken out and replenished with an equal volume of fresh media. The relative amount of siRNA released was determined by using agarose gel electrophoresis.

### Cell Culture and Cytotoxicity Assay

The human liver cancer cells SK-Hep1 were obtained from American Type Culture Collection and maintained in DMEM. The culture mediums were supplemented with 10% FBS, penicillin (100 U), and streptomycin (100 U). Cells were kept at 37°C in a humidified incubator with 5% CO_2_. Cell viability was measured by the MTS (Promega) assay. Cells were seeded in 96-well plates (5 × 10^3^ cells/well) and incubated with different concentrations of PCL-PDEM/QDs solutions for 48 h. MTS solution in PBS buffer was added into the cells, and the cells were incubated for 4 h at 37°C with 5% CO_2_. The cells were gently shaken for 5 min, and the absorbance was measured with a microplate reader (Epoch; Bio-Tek, USA) at a wavelength of 490 nm. The cell viability was calculated by normalizing the absorbance of the treated well against that of the control well. The cell viability was expressed as a percentage, assigning the viability of control cells as 100%.

### Transfection

The siRNA with FAM probe were used for laser scanning confocal imaging analysis and flow cytometry assay. Cells were planted onto 12-well plates in medium without antibiotics to give 30% to 50% density. For siRNA loading, according to the P/N of PCL-PDEM-siRNA at 0.1, PCL-PDEM/QDs particle (1 mg ml^−1^ PCL-PDEM, 10 µl) was mixed with siRNA (10 µM, 8 µl) with gentle vortexing, and the mixture was left undisturbed for 30 min. Before transfection, the culture medium was replaced by fresh medium without FBS, and the previously mentioned PCL-PDEM-siRNA/QDs mixture was then added to the medium with a final volume of 1 ml (10 μg ml^−1^ PCL-PDEM and 0.08 µM siRNA). In a parallel experiment, Lipofectamine 2000 (Invitrogen), a frequently reported commercial transfection reagent, was used as positive control. The transfection efficiency was observed at 4 h posttransfection by imaging and flow cytometry.

### Imaging

After being transfected for 4 h, the fluorescence images of treated cells were obtained using a laser scanning confocal microscope (LSCM) (TCS SP5, DEU; Leica). The FAM signals from siRNA were utilized to localize the siRNA. Before imaging, the culture media were removed, and cells were washed with PBS twice, fixed with formaldehyde (2%) for 10 min. Then the formaldehyde solution was removed, and the nuclei were stained with DAPI (Sigma) for 5 min. 

### Flow Cytometry

For the flow cytometry assays, cells were washed twice with PBS solution and harvested by trypsin. The FAM signals from siRNA were utilized to determine the transfection efficiency quantitatively. After trypsinization, the cells were resuspended in 500 µl PBS solutions and analyzed immediately by a flow cytometry machine (FACSAria II; BD, USA).

### Enzyme-Linked Immunosorbent Assay

The cell culture and siRNA transfection were performed as described above. After transfection for 48 h, the supernatant of culture media was collected. The content of IL-8 in supernatant was determined according to the instructions of IL-8 ELISA kit.

### Statistical Analysis

All data are presented as mean ± SD. The results were compared by analysis of variance. All statistical calculations were performed with the SPSS 11.0 software package. A *p* value less than 0.05 was regarded as statistically significant difference.

## Results and Discussion

### The Synthesis of Polymer

**Figure 1:**
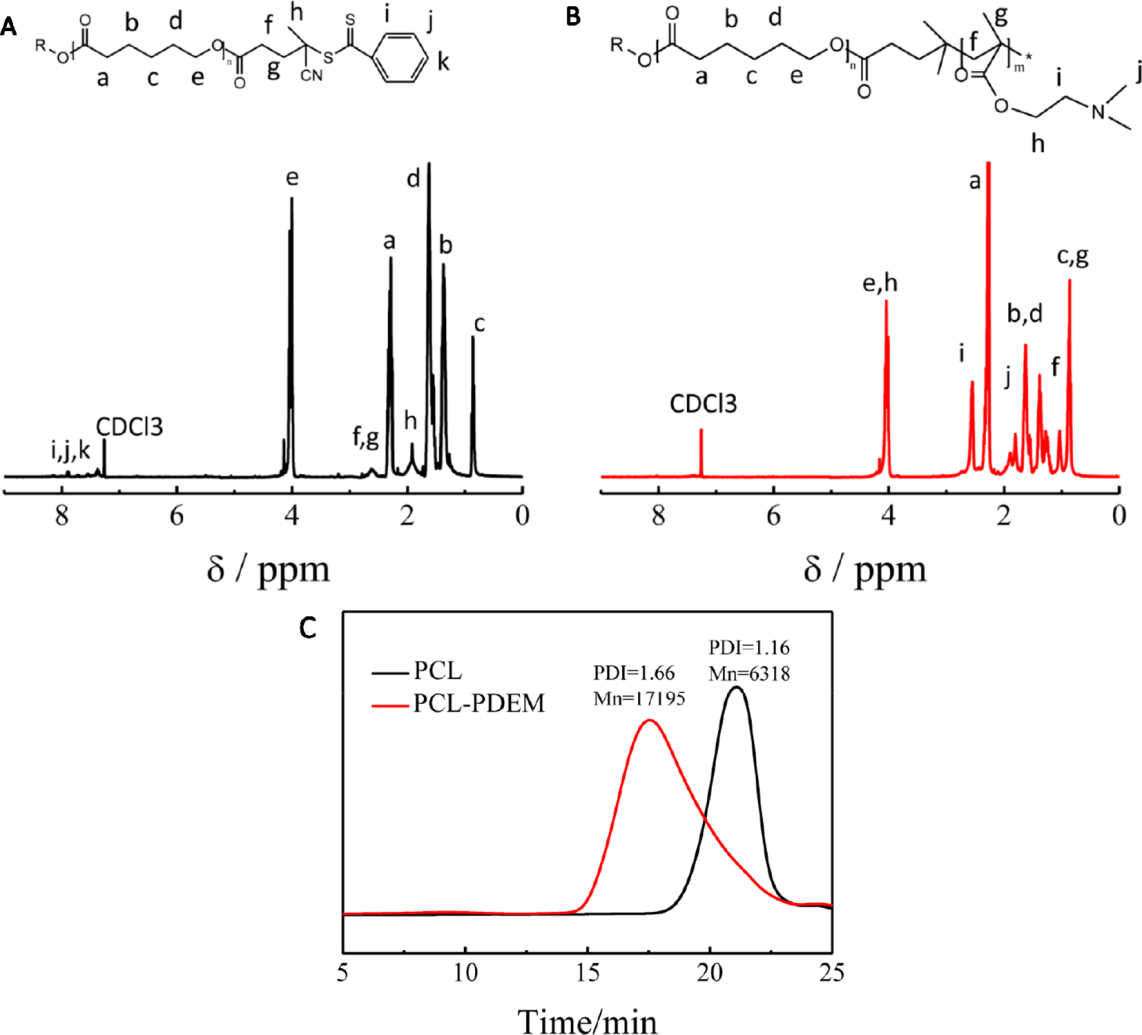
The characterization of PCL and PCL-PDEM. **(A)** ^1^H NMR of PCL-CAPD, **(B)** ^1^H NMR of PCL-PDEM, and **(C)** GPC of PCL and PCL-PDEM.

The stimuli-responsive copolymers PCL-PDEM were synthesized by RAFT polymerization of DEM using PCL-CAPD as macro RAFT agent. **Figure 1** shows the molecular information. There was a set of signals atδ7.4 to 7.9 and 2.4 to 2.6, which were assignable to CAPD that appeared in ^1^H NMR of PCL-CAPD (**Figure 1A**), indicating that the RAFT agent CAPD grafted on to PCL chains with *M*_n_ = 6318. The ^1^H NMR (**Figure 1B**) displayed clear peaks characteristic of both PDEM and PCL blocks. There were the new amino peaks of 1.80 (-NCH_2_) and 2.55 (-NCH_3_) that appeared in ^1^H NMR of PCL-PDEM. The GPC curves showed a single peak, indicating that the polymer was a block polymer with PDI = 1.66 and *M*_n_ = 17,195, rather than the mixture of PCL and PDEM. The molecular weight of PDEM block obtained from these of PCL and PCL-PDEM was 10877. These peaks indicated that PDEM had been successfully grafted onto PCL chains, which had potential to be used to prepare micelle as gene carriers.

### The Characterization of Nanoparticles

**Figure 2:**
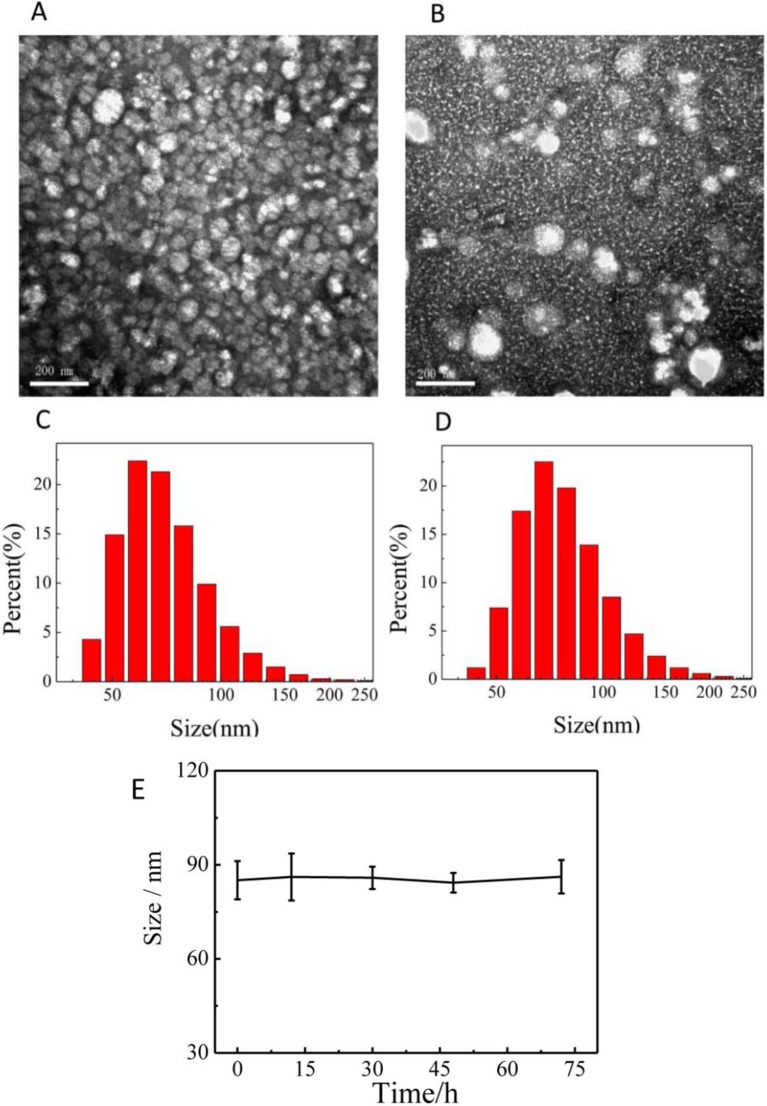
The characterization of nanoparticles dispersed in PBS. **(A)** the TEM of PCL-PDEM, **(B)** the TEM of PCL-PDEM/QDs, **(C)** DLS of PCL-PDEM, and **(D)** DLS of PCL-PDEM/QDs; **(E)** monitoring of the size change of the PCL-PDEM/QD nanoparticles in PBS at indicated time points by DLS measurement. Scale bar: 200 nm.

The morphology and diameter of prepared micelles were systematically characterized. **Figure 2** was the TEM images and particle size distributions of PCL-PDEM micelles and PCL-PDEM/QDs, respectively. The micelles had spherical structure with a particle size of about 75 ± 7 nm. The particle size of micelle increased slightly after encapsulating QDs, around 83 ± 3 nm. To assess the stability of the PCL-PDEM/QDs nanoplexes in endosome environments, the particle size of the nanoplexes was monitored in a PBS buffer (pH 5.5, 10 mM). As shown in **Figure 2E**, the size of the PCL-PDEM/QDs nanoplexes did not change during 3 days of incubation in 37°C. This result indicated that the PCL-PDEM/QDs nanoplexes had great colloidal stability in physiological endosome environments.

**Figure 3:**
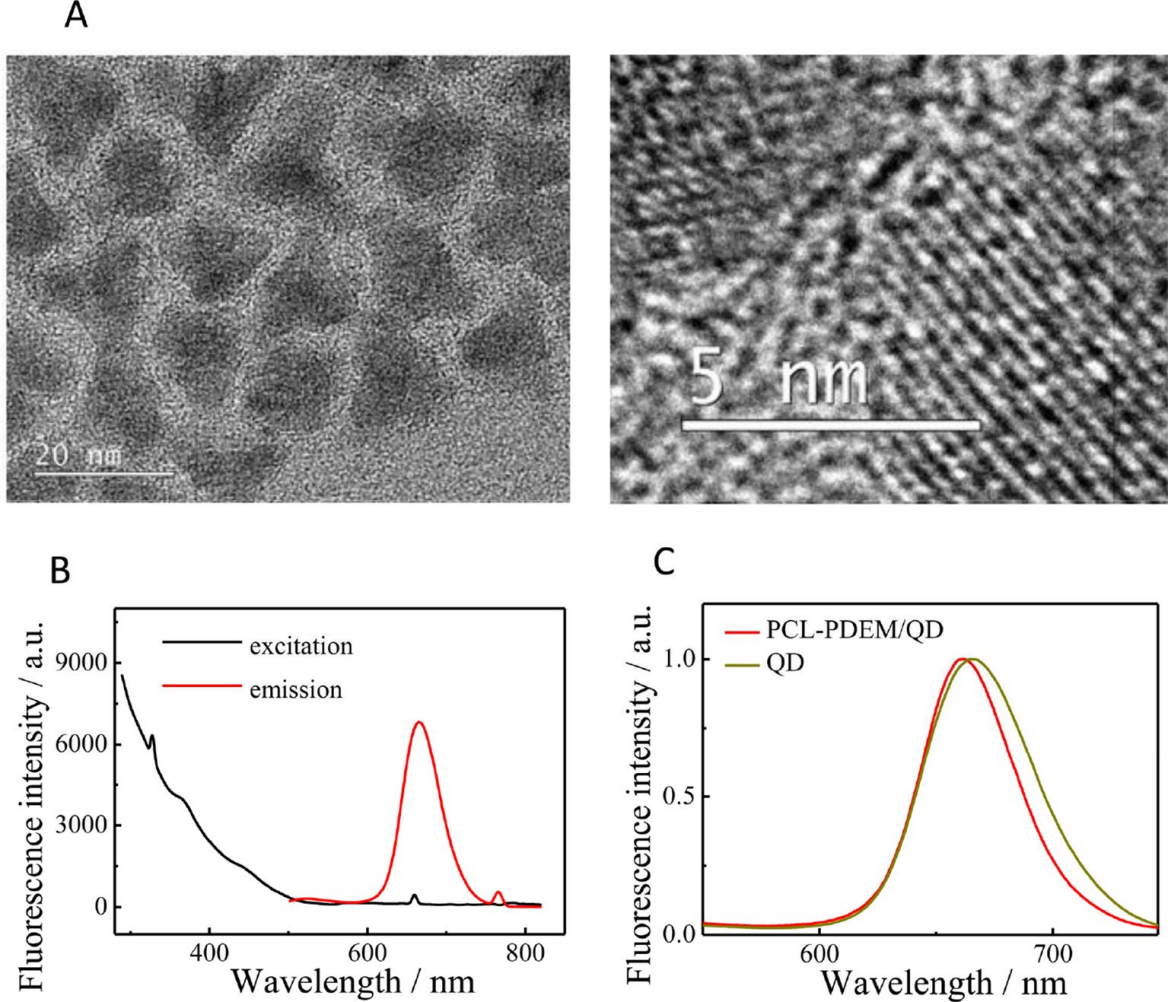
The characterization of CdSSe/ZnS QDs. **(A)** TEM of QDs, **(B)** the fluorescence spectra of QDs (0.02 mg ml^−1^ in toluene, ex: 380 nm, em: 660 nm), **(C)** the fluorescence spectra of PCL-PDEM/QD micelles.

The QDs used in this work were CdSSe/ZnS QDs. The TEM image is shown in **Figure 3A**. They were highly monodispersed with the size of about 15 nm. The fluorescence spectrum of QDs (**Figure 3B**) showed the excitation and emission spectra of the dispersion of CdSSe/ZnS QDs; 380 nm was chosen as excitation wavelength. The QDs exhibit an emission peak at 660 nm. As shown in **Figure 3C**, it can be seen that the fluorescence intensity of PCL-PDEM/QD micelles is about 800, and the emission wavelength is 660 nm when the excitation light is 380 nm. Compared with naked QDs, the emission peak of PCL-PDEM/QD micelles blue-shifted slightly due to the corrosion of zinc sulfide on the surface of QDs by buffer during the preparation (Yang et al., 2013).

**Figure 4:**
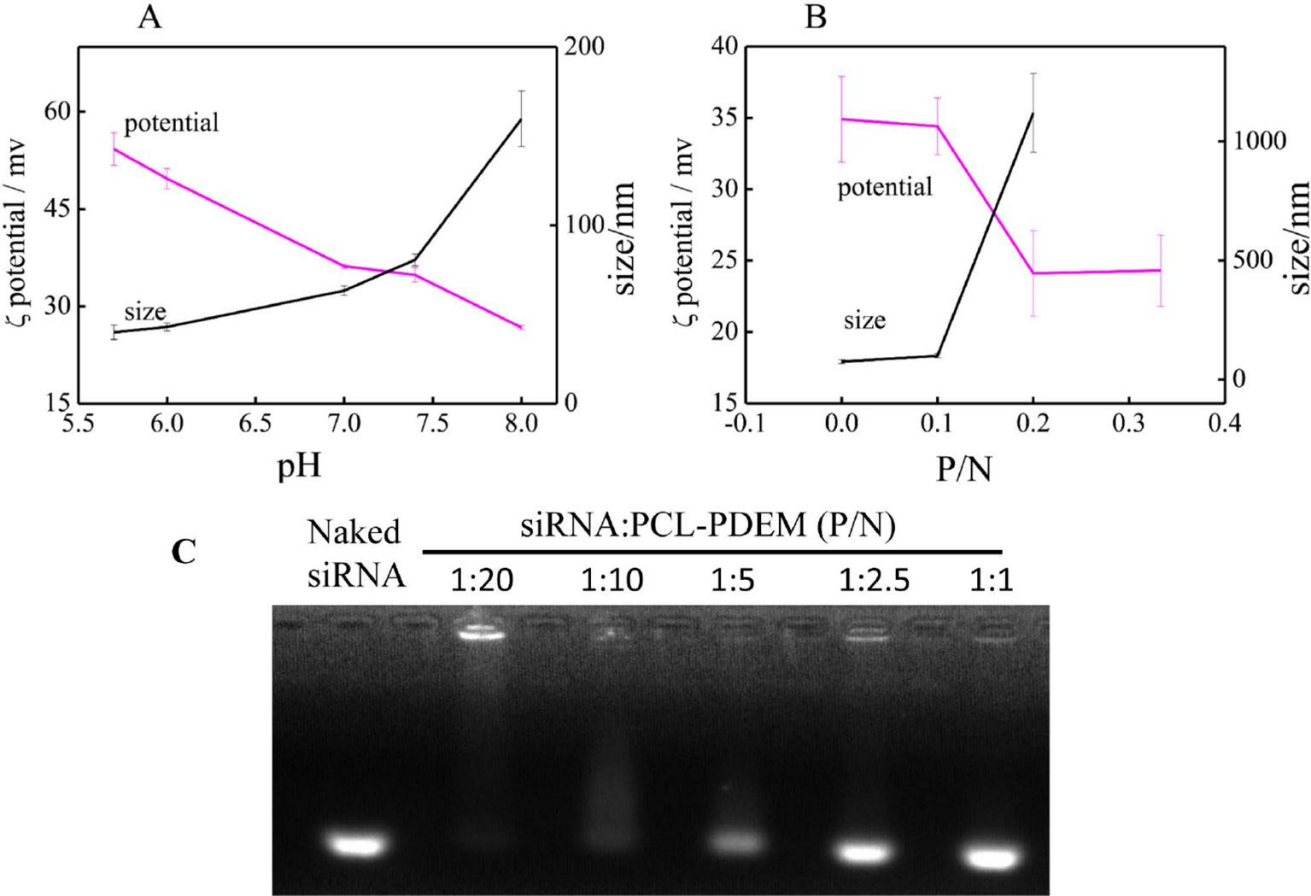
The characterization of nanoparticle. **(A)** The pH response of PCL-PDEM gene carrier. **(B)** The zeta potentials and particle sizes of PCL-PDEM/siRNA complexes with different P/N molar ratios. **(C)** Agarose gel electrophoresis of the PCL-PDEM/siRNA nanoplexes with different P/N ratio.

**Figure 4A** shows the particle sizes and zeta potentials of PCL-PDEM gene carriers in 0.01 M PBS buffer solution with different pH. The sample concentration is 1 mg ml^−1^. Due to the ionization of amines groups on the shells, the micelles were positively charged. The particle size increased, and the zeta potential decreased according to the pH increase. It indicated that with the pH increase, the ionization of amines decreased. The positive charges of micelle in acid and the “proton sponge” effect of polymer might disrupt the endosomal membrane and facilitate the nanoparticle escape (Hu et al., 2007). The gene carriers solution with pH 7.4 was selected for the subsequent cell experiment.

Highly negatively charged IL-8 siRNAs were conjugated to the micelle gene carrier through electrostatic interaction. The ratio between siRNAs and micelle was optimized to get maximum transfection efficiency. The IL-8 siRNAs were added into PCL-PDEM micelle solution according to the molar ratio of phosphoric acid group and amino group (P/N). After mixing, the zeta potentials and particle sizes of PCL-PDEM/siRNA complexes were measured and showed in **Figure 4B**. It was found that zeta potential decreased and the particle size increased with the increase in P/N ratio, which demonstrated that the charge on the amino groups of micelles was neutralized, and the siRNAs were conjugated with the micelles. However, the micelles aggregated when the ratio of P/N was more than 1:10. As shown in **Figure 4C**, when the P/N of PCL-PDEM/siRNA was greater than 1:5, the binding efficiency of PCL-PDEM was very low due to the considerable amount of free siRNA escaping from micelle. However, by decreasing the P/N to 1:10, the siRNA was observed to be completely bound with micelle. Considering the delivery efficiency, we applied the ratio of P/N at 1:10 to perform the subsequent cell experiments.

The release of nucleic acid from the nucleic acid/polycation nanoplexes is induced after nucleic acid dissociation from the polycation (Itaka and Kataoka, 2009). Inside the cells, the dissociation of nanoplexes is considered to take place by an interexchange reaction of the complexed nucleic acid with the surrounding polyanion, such as cytoplasmic mRNA, phosphatidylserine, or anionic proteoglycan (LabatMoleur et al., 1996; Itaka et al., 2004). By using the doubly labeled (fluorescein and X-rhodamine) pDNA and poly(aspartic acid) as the model polyanion, Katayose and Kataoka (1997), Itaka et al. (2002), and Itaka et al. (2004) studied the interexchange reaction between the polyanion and the complexed pDNA in the LPEI, BPEI, and PLL/pDNA nanoplexes by the fluorescence resonance energy transfer measurement. The study showed that the release of DNA was induced by the chain exchange reaction of the complexed pDNA with poly(aspartic acid).

**Figure 5:**
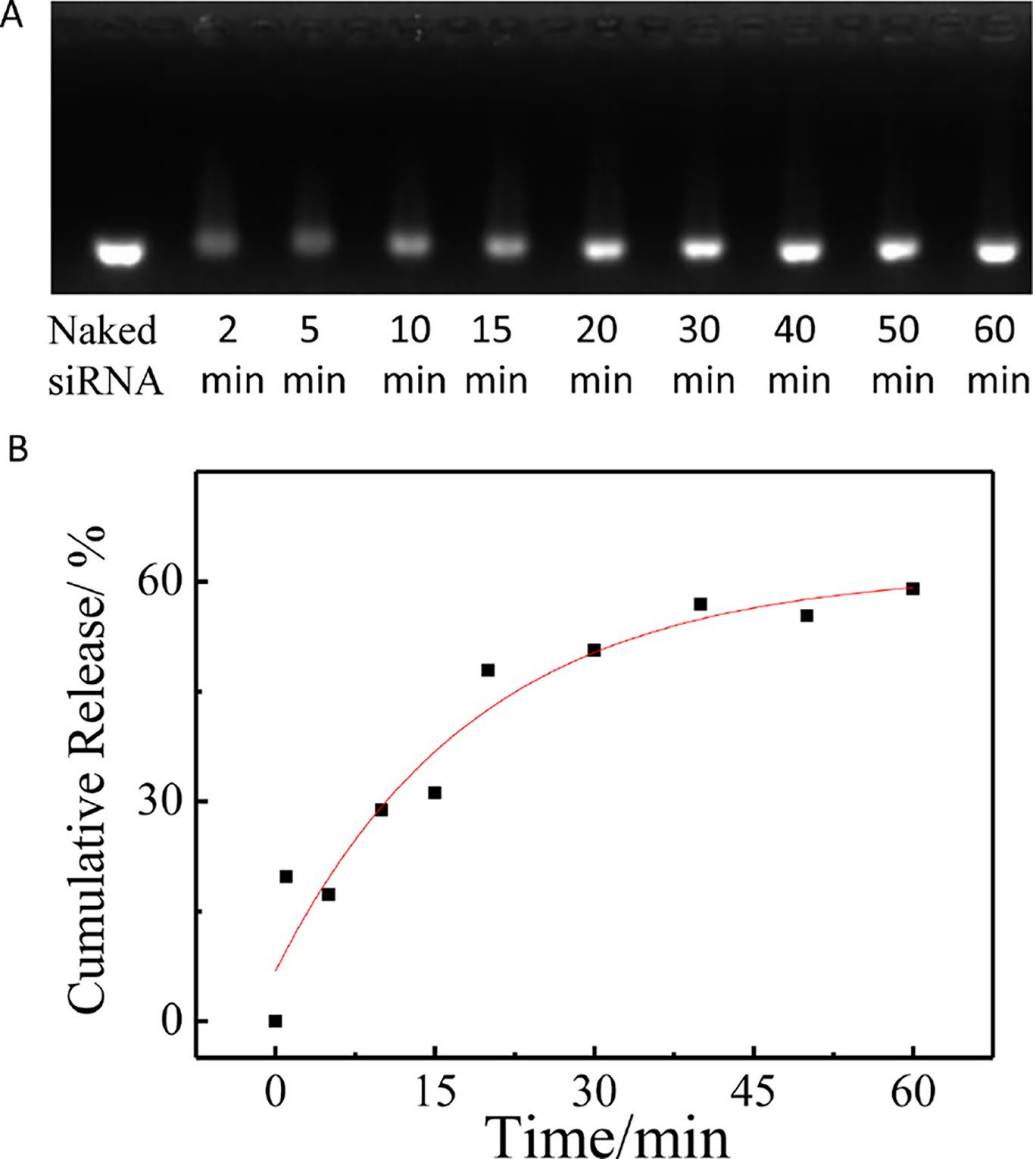
The release of siRNA from PCL-PDEM/siRNA nanoplexes in the presence of poly(aspartic acid). **(A)** Agarose gel electrophoresis of the PCL-PDEM/siRNA nanoplexes after dialysis with different minutes. **(B)** The cumulative release of siRNA.

Here, the siRNA release studies were carried out in PBS (pH 7.4, 10 mM) and 37°C using a dialysis tube (MWCO 100000) in the presence of poly(aspartic acid). As shown in **Figure 5**, as time prolonged, the free siRNA showed an obvious increase, which indicated that the siRNA decondensation was induced by the dissociation from polycation micelles due to the chain exchange reaction of the complexed siRNA with poly(aspartic acid).

### Transfer SiRNA and QDs Into Cells by Stimulus-Responsive Gene Carriers

**Figure 6:**
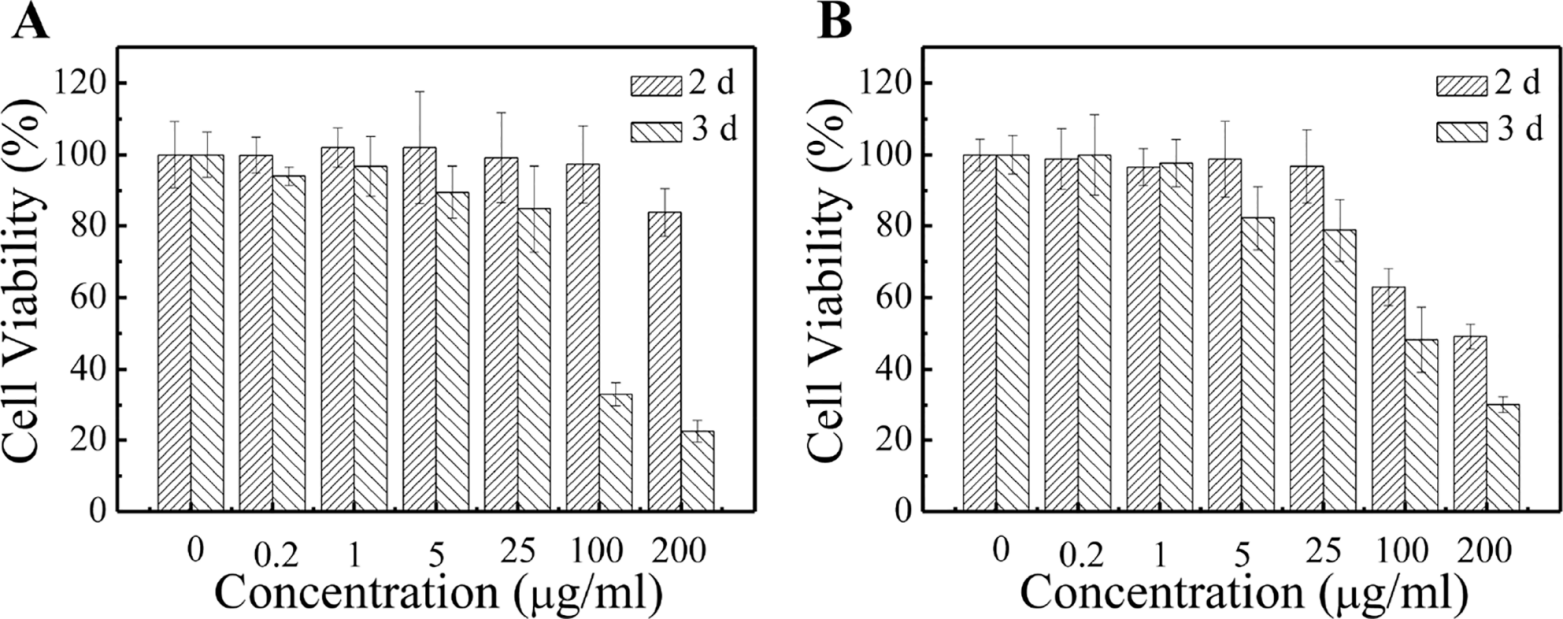
Cytotoxicity tests of PCL-PDEM/QD formulation by MTS assays. **(A)** Sk-Hep1 cells and **(B)** L929 cells.

In order to determine the safe dosage of the PCL-PDEM/QDs formulation, *in vitro* cell viability studies were carried out by using MTS assay. The hepatoma cells SK-Hep1 and L929 cells were treated with various doses of PCL-PDEM/QDs for 48 and 72 h. **Figure 6** shows that the cell viability decreased with the increase of PCL-PDEM/QD concentration. The cell viability was greater than 80% when the applied doses ranged from 0.2 to 25 μg ml^−1^. This indicated that the prepared micelle-encapsulated QDs were nontoxic on the SK-Hep1 cells if the concentration of the nanoplex was less than 25 μg ml^−1^ (higher than transfection concentration), and biomedical applications could be applied safely.

**Figure 7:**
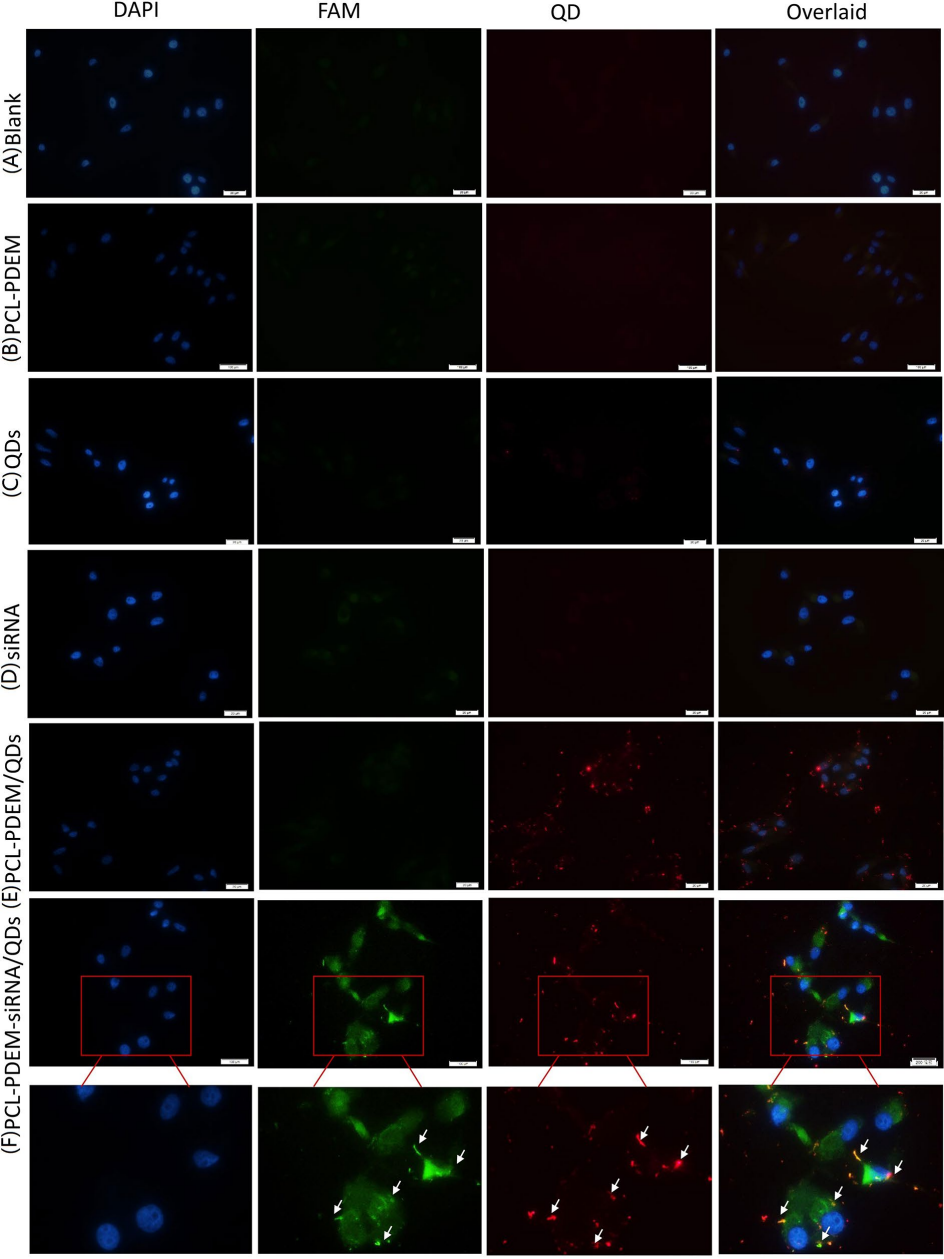
The fluorescence images of cells treated with different nanoformulations for 4 h. **(A)** Blank, **(B)** PCL-PDEM, **(C)** QDs, **(D)** PCL-PDEM/QDs, **(E)** siRNA, and **(F)** PCL-PDEM-siRNA/QDs complex into cell medium. The P/N molar ration in complex is 1:10. Scale bar: 50 µm.

The PCL-PDEM micelles were then served as nanocarriers to deliver siRNA, which target human IL-8 gene. The human hepatoma cell SK-Hep1 was used as a model. **Figure 7** showed the images of SK-Hep1 cells treated with different formulations for 4 h, where the siRNAs were labeled with fluorescent FAM for localization. As shown in **Figures 7C, D**, cells treated with PCL-PDEM/QDs showed stronger red signal than those treated with naked QDs, which indicated that the micelle-encapsulated QDs were more easily entered to cells compared to naked QDs. In **Figure 7E**, no green FAM fluorescence signal was detected in the cells treated with the naked IL-8 siRNAs labeled with fluorescence FAM, which suggested that the free siRNA was unable to penetrate the cell membrane in the absence of carriers. It was because of the fact that the uptake of negatively charged siRNA was impeded by the cell membrane. However, as shown in **Figure 7F**, the FAM fluorescent signal from IL-8 siRNA was detectable in cells treated with PCL-PDEM-siRNA formulation at the same dosage. It indicated that PCL-PDEM–loaded siRNAs could undergo uptake by cells. The uptake of polymeric micelles by the cells is affected by the surface charge and functional groups of micelles (Amjad et al., 2017). The nanocarrier used in this study was cationic micelles, so the uptake of the micelles was due to the electrostatic interaction between micelles and cells. These results demonstrated that the positively charged gene carrier successfully transports QDs and siRNA across the cancer cells.

As shown in **Figure 7**, there was a dark green fluorescent spot (siRNA^FAM^) near each red spot (QDs), indicating that the siRNA and QDs were codelivered into cells successfully. The nanoparticles had the potential for long-term real-time monitoring of the siRNA delivery process* in situ* due to the exceptional optical properties of QDs’ high resistance to photobleaching.

**Figure 8:**
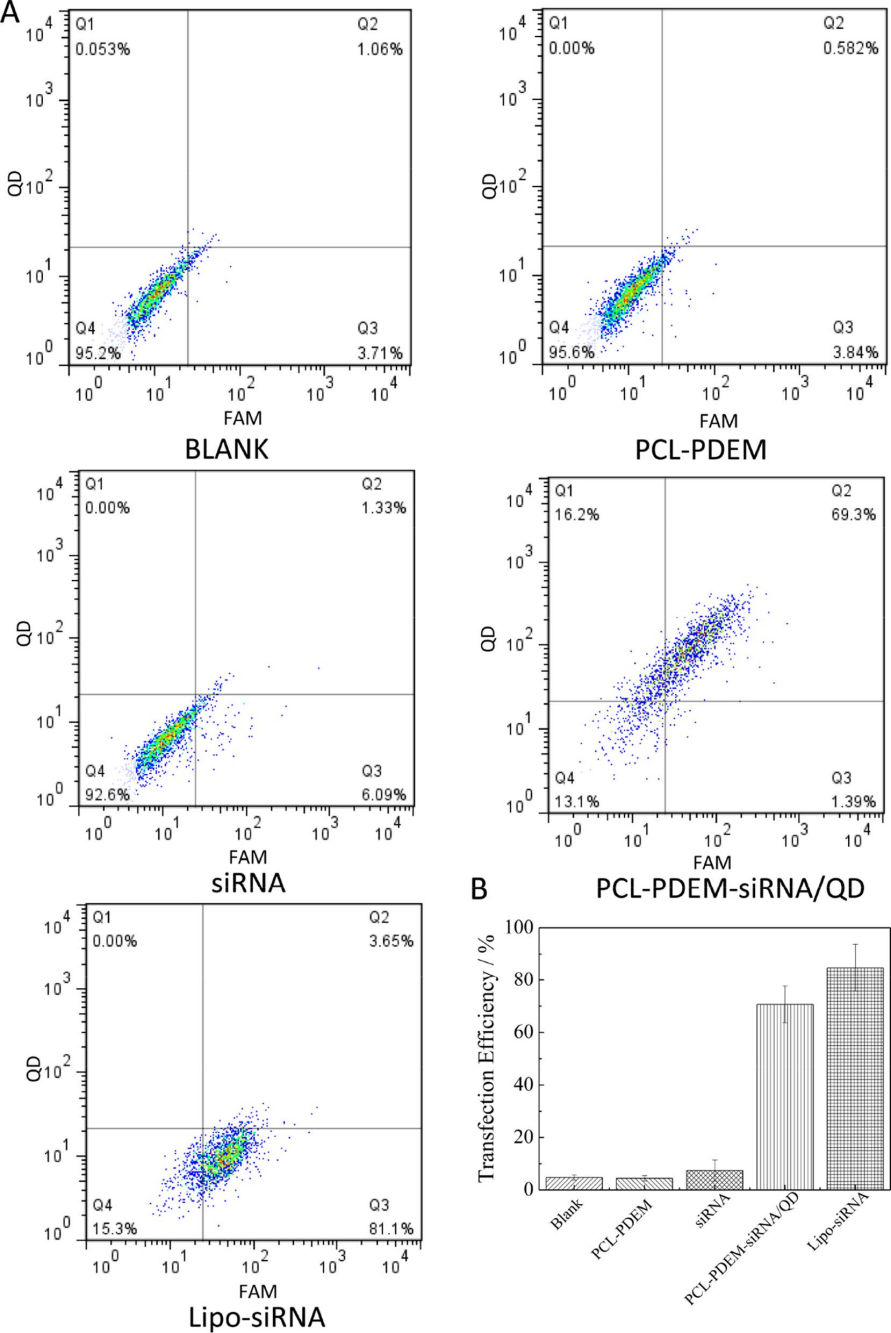
Transfection efficiency of SK-Hep1 cells different nanoformulations determined by flow cytometry analysis. **(A)** Representative pictures, where cells were treated with (I) blank, (II) PCL-PDEM, (III) free siRNA, (IV) PCL-PDEM-siRNA/QDs, and (V) Lipo-siRNA. **(B)** Percentage of cells transfected after 4-h treatment, evaluated from experiments shown in **(A)**. Values are mean ± SD, n = 3.

In order to further quantitatively evaluate the transfection efficiency of siRNA by PCL-PDEM/QDs nanocarriers, the flow cytometry assay was performed. As shown in **Figure 8**, the result shows that no fluorescence signals were detected in the cells treated with naked IL-8 siRNA^FAM^, consistent with the imaging analysis, which indicated that naked siRNA could not enter into cells. In contrast, the Lipofectamine 2000 (Lipo), a commercial siRNA transfection reagent, was used to deliver siRNA^FAM^ as the positive control group. The strong fluorescence signals of QDs and FAM were detected in the cells treated with PCL-PDEM-siRNA/QDs, which suggested quantitatively the siRNA^FAM^ and QDs were delivered successfully into the tumor cells. The fraction of double-positive fluorescence signals for the tumor cells treated with PCL-PDEM-siRNA/QDs was about 69.3%. And the fraction of FAM-positive signals for the cells treated with PCL-PDEM-siRNA/QDs and Lipofectamine 2000 were about 71% and 85%, respectively. Although the nanocarrier had comparable transfection efficiency compared with the Lipofectamine 2000 transfection reagent, the nanocarrier could load hydrophobic QDs, which were common and commercially available due to hydrophobic core of nanocarrier, and realize codelivery of siRNA and QDs. However, the Lipofectamine 2000 could not load hydrophobic QDs. These results demonstrated that the micelle-encapsulated QDs can be utilized as efficient siRNA delivery nanocarriers.

**Figure 9:**
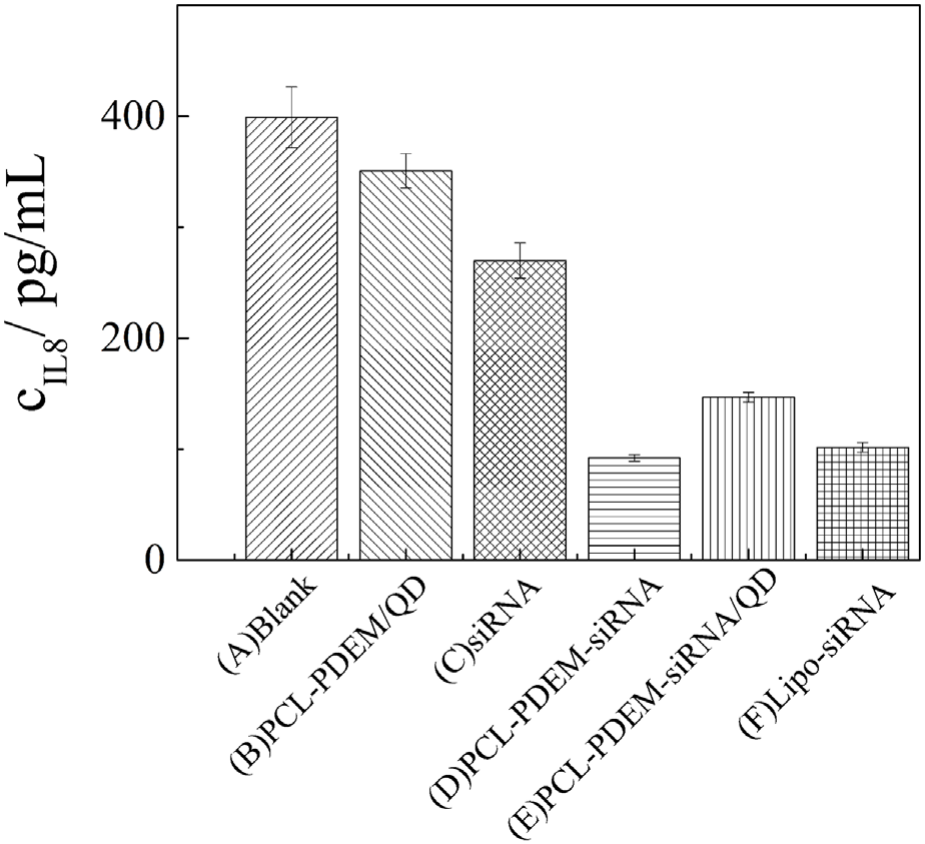
IL-8 expression levels in SK-Hep1 cells were assayed by ELISA. **(A)** Blank, **(B)** PCL-PDEM/QDs, **(C)** siRNA, **(D)** PCL-PDEM-siRNA, **(E)** PCL-PDEM-siRNA/QDs, and **(F)** Lipo-siRNA. Values are mean ± SD, n = 3.

In addition, after SK-Hep1 cells were treatment with PCL-PDEM-siRNA nanoplex for 48 h, the IL-8 expression levels in culture media were assayed, and the gene silencing efficiency of nanoplex was evaluated. The results in **Figure 9** showed that a remarkable decrease of IL-8 expression was observed from the cells treated with PCL-PDEM-siRNA, PCL-PDEM-siRNA/QDs, and Lipo-siRNA, compared to the other groups. The suppression of IL-8 expression for PCL-PDEM-siRNA/QDs was about 63% after 48 h of treatment, slightly less than that for Lipo-siRNA, which was about 75%. It demonstrated that the formulation of PCL-PDEM-siRNA/QDs had great potential for gene therapy applications.

## Conclusions

In summary, we have proposed a pH stimuli-responsive micelle based on PCL-PDEM copolymer to deliver siRNA with high transfection efficiency for hepatoma cancer therapy. The properties of the nanoparticles have been extensively analyzed. A specific siRNA sequence targeting IL-8 gene has been conjugated with PCL-PDEM for RNAi on SK-Hep1 cancer cells. The results showed that the transfection efficiency of the PCL-PDEM-siRNA nanoplex was about 70%, and the suppression of IL-8 expression was about 63% after 48-h treatment. The codelivery of QDs and siRNA has been realized, which is beneficial to visualize the process of siRNA delivery. No cytotoxicity of nanoplex was detected according to the MTS assays. The nanoplex formulation had potential for clinical applications in hepatoma cancer therapy.

## Data Availability Statement

The datasets analyzed in this manuscript are not publicly available. Requests to access the datasets should be directed to xugaixia@szu.edu.cn.

## Author Contributions

ZC, GX, XW, GL, and K-TY designed experiments. ZC, HX, and LL carried out experiments. ML and PZ assisted with sample collecting. ZC, GX, GL, XW, and K-TY analyzed experimental results. ZC wrote the manuscript, and GX revised the manuscript. All authors have contributed to the final version and approved the final manuscript.

## Funding

This work was supported by the National Natural Science Foundation of China (31671491, 21677102, 81772002), China Postdoctoral Science Foundation (2017M612719), Natural Science Foundation of Guangdong Province (2018A030310415), and the Basic Research Foundation of Shenzhen (JCYJ2017081710263496).

## Conflict of Interest

The authors declare that the research was conducted in the absence of any commercial or financial relationships that could be construed as a potential conflict of interest.
